# Knowledge Tracing via Attention Enhanced Encoder-Decoder

**DOI:** 10.1155/2022/1552745

**Published:** 2022-12-17

**Authors:** Kai Zhang, Zhengchu Qin, Ying Kuang

**Affiliations:** ^1^School of Computer Science, Yangtze University, Jingzhou 434000, China; ^2^School of Foreign Languages, Yangtze University College of Arts and Sciences, Jingzhou 434020, China

## Abstract

The knowledge tracing model takes students' learning behaviours data as input to determine their current knowledge status and predict their future answers. The learning behaviours data describes three main types of learning behaviours: learning process, learning end, and learning interval. The classical knowledge tracing models only use the data of the learning end, which contains limited information and the models cannot accurately describe constraint in the same learning behaviour in the time series. Subsequent models add other types of learning behaviours data but do not integrate different types of learning behaviours, and the models cannot accurately describe collaboration in different learning behaviours. To address these issues, knowledge tracing via attention-enhanced encoder-decoder is proposed to synthesize and analyse the three types of learning behaviours mentioned above and firstly adopts the multiheaded attention mechanism to describe constraint in the same learning behaviours; secondly adopts the channel attention mechanism modelling collaboration in the three types of learning behaviours. In the experiments, various comparisons are made with related models on several real data sets, and the results show that our model achieves certain advantages in terms of performance and knowledge state representation. In terms of practical application, an intelligent learning platform based on the model has been implemented, which predicts the future answer of students in the teaching process of two offline courses: computer and English and has achieved better performance than other knowledge tracing models.

## 1. Introduction

Influenced by the COVID-19 epidemic, the public gradually accepts smart education platforms such as Intelligent Tutoring System (ITS) and Massive Open Online Course (MOOC). However, the initial endowment attributes of smart education do not include functions such as determining the state of students' knowledge and predicting their future learning performance.

For these reasons, Knowledge Tracing (KT) has become an important research element in the field of smart education, which analyses the learning behaviours data collected by the platform to determine the students' knowledge status and predicts their future performance in answering exercises based on the knowledge status. Knowledge tracing is now widely used in various online education platforms, such as Academy Online, Khan Academy, edX, Coursera, and so on. The main meaning and function of current knowledge tracing is to provide fine-grained educational strategies for smart education platforms by grasping students' knowledge status and predicting future performance of answering questions and to provide personalized educational services for each student.

**Figure 1:**
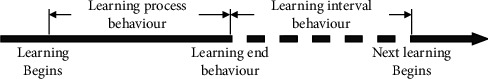
Learning behaviours and their sequential relationship.

Learning sequences consist of students' learning records, mainly those of students' learning behaviours. Learning behaviours data can be generally divided into three categories [1], namely learning process data, learning end data, and learning interval data, which are used to describe the corresponding learning behaviours in the learning records. The learning process data describe the learning process behaviour, mainly including the number of attempts to answer and the number of requests for hints. The learning end data describe the learning end behaviour, mainly including the exercises students answer and the results of their answers. The learning interval data describe the learning interval behaviour, mainly including the time interval between two adjacent learning sessions and the number of times students learn a concept.  Figure 1 shows the learning process behaviour, learning end behaviour, and learning interval behaviour and their sequential relationships.

The classical knowledge tracing models [2–4] only use the learning end data. These models are generally able to determine the basic knowledge state of students by analysing their learning end behaviour, but since the learning end data only contain information about students' correct or incorrect answers to a certain exercise, they cannot trace students' knowledge state more accurately. For example, when learning the third-person singular concept in English, students A and B have the same learning end data but different learning process data, the different knowledge states of students A and B on the third-person singular concept cannot be represented in such classical knowledge tracing models.

Students' learning records also include learning process behaviour and learning interval behaviour, which also map changes in students' knowledge states. Some researchers have used learning process data and learning end data to trace students' knowledge states [5] and learning interval data to model students' forgetting behaviours [6, 7], but none of them have considered collaboration in different behaviours, i.e., the interaction of multiple types of learning behaviours in a learning sequence.

In order to more accurately trace the state of students' knowledge, the main tasks of this paper are as follows:

1. Describing constraint in the same behaviours. First, the set of three types of learning behaviour data is selected as the input; second, the attention weights of the input data are obtained using the multiheaded attention mechanism to represent the constraint relationship of a single type of learning behaviour on the time series, which is used to describe constraint in same behaviours.
2. Describe the collaboration in different behaviours. First, the set of three types of learning behaviours data is stitched as input; second, the global information of the three types of learning behaviours is obtained using the channel attention mechanism; finally, the global information is mapped into attention weights among learning behaviours, which represent the interaction of multiple types of learning behaviours and is used to describe collaboration in different behaviours.
3. Knowledge tracing via attention-enhanced encoder-decoder is proposed. First, the encoder is used to fuse the constraint in the same behaviours and the collaboration in different behaviours; second, the decoder is used to obtain students' learning vectors and forgetting vectors by inputting different query vectors; finally, the purpose of tracing students' knowledge states more accurately is achieved.

## 2. Related Work

### 2.1. Knowledge Tracing

#### 2.1.1. Knowledge Tracing Models Based on Learning End Behaviour

Bayesian Knowledge Tracing (BKT) [2] first introduced the concept of knowledge tracing and used a probabilistic calculation method to solve the task of knowledge tracing. BKT takes the learning end data as input and defines the probability of initially learning a concept *P*(*L*_0_), the probability of transferring an unlearned state to a learned state *P*(*T*), the probability of not mastering a concept but guessing correctly *P*(*G*), the probability of the probability of mastering a concept but answering incorrectly *P*(*S*), etc., and the Hidden Markov Model (HMM) [8] is used to model the relationship between the above four probabilities to predict students' future learning performance.

Deep Knowledge Tracing (DKT) [3] first used deep sequential models to solve the task of knowledge tracing. Similar to BKT, DKT still uses learning end data as input to represent students' knowledge states with the hidden states of Recurrent Neural Network (RNN) [9] or Long Short-Term Memory (LSTM) [10], and finally a fully connected layer to predict students' future learning performance.

Dynamic Key-Value Memory Networks (DKVMN) [4], inspired by standard memory enhancement networks [11], proposes a memory matrix approach to solve the task of knowledge tracing. DKVMN still uses learning end data as input, a key matrix to store concepts, and a value matrix to store the student's mastery state of the concept; the model uses these two matrices to determine the student's mastery state of each concept at each learning session and finally outputs the probability of the student's future learning performance in a fully connected layer.

In subsequent studies, researchers still modelled students' knowledge states using only learning end data as the input to the model: Kaser et al. [12] proposed a dynamic Bayesian knowledge tracing model based on BKT to model the dependencies between different concepts; Su et al. [13] added exercise information to the input of the model based on DKT; Abdelrahman et al. [14] used a Hop-LSTM network structure on top of DKVMN, enabling the model to capture long-term boundedness in student learning records. Other variants of such models include TLS-BKT [15], Multigrained-BKT [16], PDKT-C [17], HMN [18], and other models.

BKT, DKT, and DKVMN are classical knowledge tracing models, and these models have laid a solid foundation for the subsequent studies. Their shortcomings are that only learning end data are used to trace students' knowledge states, modelling constraint in the same behaviours. Learning process data and learning interval data are not used, not modelling collaboration in different behaviours, so they cannot provide more adequate support for representing students' knowledge states.

#### 2.1.2. Knowledge Tracing Models Based on Learning Interval Behaviour

Some of the studies used learning interval data: Nagatani et al. [6] were inspired by the Ebbinghaus forgetting curve [19] and added learning interval data as input to the DKT model. They considered learning interval data as a factor affecting forgetting behaviours and were able to model forgetting behaviours by adding learning interval data as input to the model. Inspired by the memory traces of decline said [20], the study by Li et al. [7] proposed the LFKT model, which considered not only the above learning interval data but also the effect of students' conceptual mastery status on forgetting.

Although these two models add learning interval data to the use of learning end data and achieve better results, they still model only constraint in same behaviours and neglect to model collaboration in different behaviours.

#### 2.1.3. Knowledge Tracing Models Based on Learning Process Behaviour

Some of the studies used learning process data: Cheung and Yang [5] input the learning process data to a Classification And Regression Tree (CART) to predict whether students could answer the exercises correctly, then combined the predicted results with the real results, and finally input the combined data and the learning end data to DKT The combined results are then combined with the real results, and finally the combined data and the learning end data are fed into the DKT model to predict their future answers. This method uses the learning process data as a complement to the learning end data to improve the method of modelling constraint in the same behaviours but does not yet model collaboration in different behaviours.

In general, most studies use only learning end data as input or introduce multiple types of learning behaviours data as input when tracing students' knowledge states, but none of them model collaboration in different behaviours. To address the above problems, this paper proposes a model: knowledge tracing via attention enhanced encoder-decoder, which models collaboration in different behaviours while modelling constraint in the same behaviours to provide a more adequate support for representing students' knowledge states.

### 2.2. Attentional Mechanisms

A biological perspective on attention mechanisms is based on the principle that humans selectively direct the focus of their attention based on nonvolitional cue and volitional cue [21]. Nonvolitional cue refers to the fact that a person is not cognitively and consciously driven to access information, and volitional cue refers to the fact that a person is cognitively and consciously driven to access information. In attention mechanisms, queries refer to volitional cues, keys, and values refer to nonvolitional cues. The benefit of adding volitional cues is to bias the output of the attention mechanism towards certain input data, rather than taking in the input data wholesale.

For example, in determining the state of students' knowledge, student S answered correctly the exercise about the concept of third-person singular in learning English. If there is no cognitive and consciousness drive and only the learning end data is used as the criterion, the teacher's attention is guided by the non-volitional cue and judges the mastery status of student S on the concept of third-person singular; however, if there is a cognitive and consciousness drive, on top of the learning end data, the teacher will also notice the learning process data and learning interval data of the student. The teacher's attention is guided by the volitional cue to judge the state of student S's mastery of the third-person singular concept.

Ghosh et al. [22] proposed the AKT model to solve the knowledge tracing task by constructing context-aware representations of exercises and outcomes and summarizing students' past performance using attention mechanisms. The inputs of the attention mechanisms are query, key, and value, and the output is a weighted sum of values, and the attention weights are obtained by calculating the similarity of query and key. The self-attention mechanism is a variant of the attention mechanism, which has inputs from the same data and is better at capturing the similarity within the data and reduces the dependence on external data because there is no input from external data. Pandey et al. [23] proposed the SAKT model, which first applied the Transformer model [24] to the domain of knowledge tracing by describing the inputs in terms of temporal constraint relations to solve knowledge tracing tasks. The main structure of the Transformer model is a multiheaded attention mechanism, consisting of multiple attention mechanisms or self-attention mechanisms in parallel, where a fully connected layer maps the input data to different subspaces and is able to learn different weights based on the same mechanism, which is used to describe constraint in same behaviours.

The disadvantage of the multiheaded attention mechanism using learning process data, learning end data, and learning interval data as volitional cues is that different learning behaviours are treated as having the same weight when tracing knowledge states. The channel attention mechanism solves this problem [25–28] by using three types of learning behaviours data as input to the channel attention mechanism, the “squeeze” operation collects global information about the three types of learning behaviours data, and the “stimulate” operation converts the global information into attention weights, which represent the interaction of multiple types of learning behaviours, are used to describe collaboration in different behaviours.

## 3. Materials and Methods

### 3.1. The Idea Proposed by the Model

As a whole, the learning sequence includes several different types of learning behaviours, such as the learning process behaviour, the learning end behaviour, and the learning interval behaviour. In this paper, we use learning process data *b*^*I*^, learning end data *b*^*II*^, and learning interval data *b*^*III*^ to describe the above three types of learning behaviours, in which *b*^*I*^ mainly includes data such as the number of attempts of students to answer and the number of requests for hints; *b*^*II*^ mainly includes data such as the exercises students answer and the results of their answers; *b*^*III*^ mainly includes data such as the time interval between two adjacent learning sessions and the number of times students learn a concept. This paper finds that the learning behaviours possess the constraint in the same behaviours and collaboration in different behaviours. The specific descriptions are as follows:

According to the literature [29], changes in students' knowledge states are bounded by their pre-existing knowledge states and are manifested in the constraint in the same behaviours, i.e., changes in knowledge state are reflected in a learning behaviour is gradual. Specifically, the constraint in the learning process data *b*^*I*^ may be manifested by the fact that the change in the number of attempted responses for a given exercise is smooth at adjacent time steps; the constraint in the learning end data *b*^*II*^ may be manifested by the fact that the change in the result of a student's response to a given exercise is also smooth; the constraint in the learning interval data *b*^*III*^ may be manifested by the fact that the change in a number of adjacent learning intervals is also flat. From a modelling perspective, the characterization of the three types of learning behaviours data should take into account their respective similarity constraints to reflect the objective changes in students' knowledge states, which are neglected in the current study.

According to the literature [30], the interaction of multiple types of learning behaviours in a learning sequence is manifested by the collaboration in different behaviours. Specifically, the collaboration in learning process data *b*^*I*^ and learning end data *b*^*II*^ may be manifested in that the probability of correct answers for a given exercise is lower when students have more attempts and higher when they have fewer attempts; the collaboration in learning interval data *b*^*III*^ and learning end data *b*^*II*^ may be manifested in that the probability of correct answers for a given exercise is lower when students have a longer learning interval and higher when they have a shorter learning interval. From the modelling point of view, the collaboration in different learning behaviours data should be considered in order to reflect the objective changes of students' knowledge state, which is neglected in the current study.

BKT uses learning end data *b*^*II*^ to trace the student's knowledge state. However, because *b*^*II*^ only contains information about students answering a certain exercise correctly or incorrectly, and the model does not express constraint in learning end behaviour on the time series. Although subsequent studies [12–15] still used only the learning end data *b*^*II*^, they mostly used deep models, so there was some progress in modelling the boundedness of the learning end behavior. Subsequently, some researchers added learning process data *b*^*I*^ [5] and learning interval data *b*^*III*^ [6, 7] to the input of the model to improve the performance of the model. Although these studies validated the validity of other learning behaviours, they did not model collaboration in different behaviours in a learning sequence.

In summary, it is advantageous to integrate multiple types of learning behaviours data when tracing students' knowledge states, which enables knowledge tracing models to more accurately predict students' future performance. However, when modelling learning behaviours, the constraint in the same behaviours and collaboration in different behaviours should be considered in an integrated manner.

In this paper, we use the multiheaded attention mechanism to adaptively assign the weights of each type of learning behaviours data itself, so as to model constraint in the same behaviours; and the channel attention mechanism to adaptively assign the weights between different types of learning behaviours data, so as to model collaboration in different behaviours.

### 3.2. Definition of Learning Behaviours Data

In this paper, we define three types of learning behaviours data as follows: learning process data *b*_*t*_^*I*^=(AN, RN, FA) describes the learning process behaviour of the student's *t*, *t* ≥ 1 th learning record, where AN ∈ *ℕ* indicates the number of times the student attempted to answer; RN ∈ *ℕ* indicates the number of times the student requested a hint; FA={0,1} indicates the first action of the student when answering the exercise, where 1 indicates that the student first attempted to answer and 0 indicates that the student first requested a hint. *B*^*I*^=(*b*_1_^*I*^, *b*_2_^*I*^, ⋯*b*_*n*_^*I*^) is the set of learning process data *b*^*I*^, i.e., it is composed of learning process data *b*_*n*_^*I*^, *n* ≥ 1 .

The learning end data *b*_*t*_^*II*^=(*q*_*t*,_*r*_*t*_) describes the learning end behaviour of the student's *t*, *t* ≥ 1 th learning record, where *q*_*t*_ ∈ *ℕ* indicates the exercise that the student answered; *r*_*t*_={0,1} indicates the result of the student's answer, where 1 indicates that the student answered the exercise correctly and 0 indicates that the student answered the exercise incorrectly. *B*^*II*^=(*b*_1_^*II*^, *b*_2_^*II*^, ⋯*b*_*n*_^*II*^) is the set of the learning end data *b*^*II*^, which is composed of the learning end data *b*_*n*_^*II*^, *n* ≥ 1.

**Figure 2:**
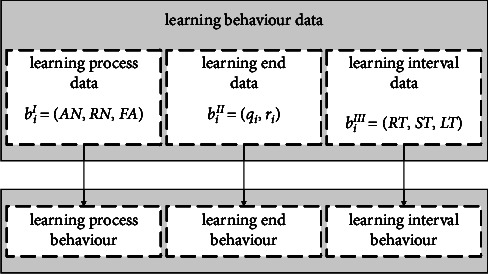
Correspondence between learning behaviours and their data.

The learning interval data *b*_*t*_^*III*^=(RT, ST, LT) describes the learning interval behaviour of the student's *t*, *t* ≥ 1 th learning record, where ST ∈ *ℕ* indicates the time interval between the student's *t* − 1 th learning and *t* th learning; RT ∈ *ℕ* indicates the time interval between the student's learning of the current concept; LT ∈ *ℕ* indicates the number of repetitions of the current concept. *B*^*III*^=(*b*_1_^*III*^, *b*_2_^*III*^,…, *b*_*n*_^*III*^) is the set of the learning end data *b*^*III*^, i.e., it consists of the learning interval data *b*_*n*_^*III*^, *n* ≥ 1.  Figure 2 shows the learning behaviours described by the learning behaviours data in the learning sequence.

### 3.3. Knowledge Tracing via Attention Enhanced Encoder-Decoder

**Figure 3:**
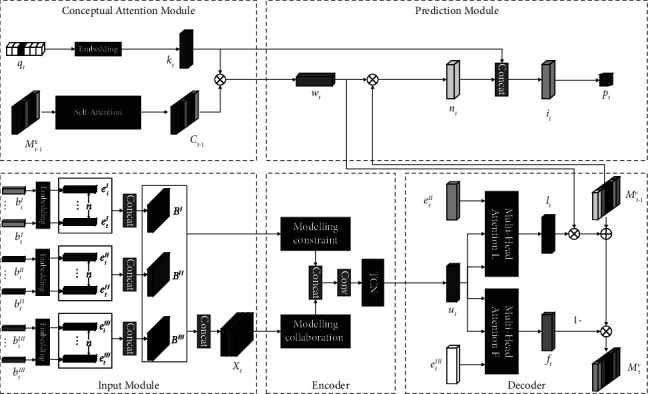
Knowledge tracing via attention enhanced encoder-decoder.

In this paper, we propose Knowledge Tracing via Attention Enhanced Encoder-Decoder (AED-KT), whose overall architecture is shown in Figure 3.

The model consists of five components: an input module, an encoder, a decoder, a conceptual attention module, and a prediction module. The input module embedding represents a number of continuous learning behaviours data. The encoder models constraint in the same behaviours and collaboration in different behaviours. The decoder generates students' learning and forgetting vectors and updates the state matrix *M*_*t*−1_^*v*^. The conceptual attention module is used to capture the similarity between concepts. The prediction module predicts students' answers at moment *t* based on the state matrix *M*_*t*−1_^*v*^, the concept matrix *M*_*t*−1_^*k*^, and the exercise *q*_*t*_, *t* ≥ 1. Concept matrix *M*^*k*^ represents the concept and state matrix *M*^*v*^ represents the student's concept mastery state, and these two matrices are dynamically updated with the learning sequence.

#### 3.3.1. Input Module

The learning process data *b*_*i*_^*I*^=(AN, RN, FA), *i* ≥ 1 is represented as a row vector: *b*_*i*_^*I*^*ϵℝ*^1×3^, and multiplied with the embedding matrix *C*^*I*^*ϵℝ*^1×3^ to obtain vector *e*_*i*_^*I*^*ϵℝ*^1×*d*_*v*_^. The learning end data *b*_*i*_^*II*^=(*q*_*i*,_*r*_*i*_), *i* ≥ 1 is transformed into one-hot encoding: *b*_*i*_^*II*^*ϵℝ*^1×2*N*^, and multiplied with the embedding matrix *C*^*II*^*ϵℝ*^2*N*×*d*_*v*_^ to obtain vector *e*_*i*_^*II*^*ϵℝ*^1×*d*_*v*_^ in order to solve the problem of *b*_*i*_^*II*^ sparsity. The learning interval data *b*_*i*_^*III*^=(RT, ST, LT), *i* ≥ 1 is represented as a row vector: *b*_*i*_^*III*^*ϵℝ*^1×3^ , and multiplied with the embedding matrix *C*^*III*^*ϵℝ*^1×3^ to obtain vector *e*_*i*_^*III*^*ϵℝ*^1×*d*_*v*_^.

The learning behaviours data with *n* consecutive embedding representations are taken, and then three matrices *B*^*I*^, *B*^*II*^ , and *B*^*III*^ of size *n* × *d*_*v*_ are combined according to the learning behaviour types as the input of the multi-headed attention mechanism; these three matrices are stitched into a three-dimensional array *X*_*t*_ of size 3 × *n* × *d*_*v*_ as the input of the channel attention mechanism, where 3 indicates that the array *X*_*t*_ contains three types of learning behaviours, and *n* indicates that the array *X*_*t*_ contains *n* consecutive learning behaviours, and *d*_*v*_ is the dimension of the vector representation of the learning behavior data.

#### 3.3.2. Encoder

The array *X*_*t*_ consists of matrices *B*^*I*^, *B*^*II*^ , and *B*^*III*^ stitched together, and each of these three matrices includes n consecutive learning behaviours data, which represent three different types of learning behaviours: learning process, learning end, and learning interval behaviour.

**Figure 4:**
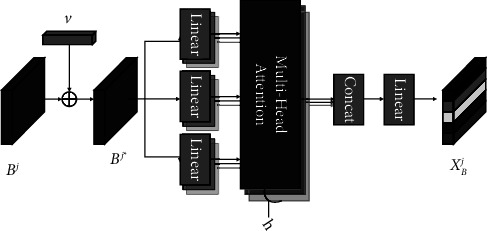
Modelling constraint in same behaviours.

*(1) Modelling Constraint in Same Behaviours*. It is important to note that each type of learning behaviour has a constraint on subsequent similar behaviours on the learning sequence, i.e., the constraint between the same type of learning behaviour in the learning sequence on the time sequence. Because the multiheaded attention mechanism can locate similar information on the learning sequence and translate it into the relative weights of the learning records in the sequence, the multiheaded attention mechanism is used to model constraint in the same behaviours, and the specific process is shown in Figure 4.

Firstly, using the parameter *v* *ϵℝ*^1×*n*^ as the position code, which represents the relative position of the data of *n* consecutive learning behaviours in time sequence, it is added to the input matrices *B*^*I*^, *B*^*II*^ , and *B*^*III*^ to form a learning behavior matrix containing relative position information in time sequence:

(1) $$\begin{array}{c} B^{j*}\left(i\right)=B^{j}\left(i\right)+v\left(i\right),j\in \left\{I,II,II\right\},i\in \left\{1,\ldots ,n\right\}\text{.} \end{array}$$

Secondly, the learning behaviour matrices *B*^*I∗*^, *B*^*II∗*^ and *B*^*III∗*^ are input into the multi-headed attention mechanism, and the attention weights are obtained by calculate the similarity between each learning behaviour, which is used to model constraint in the same behaviours, and the magnitude of the attention weights indicates the strength of the learning behaviour constraint relationship. The output matrices *X*_*B*_^*I*^, *X*_*B*_^*II*^ and *X*_*B*_^*III*^, which represent the constraint of learning process behaviour, learning end behaviour, and learning interval behaviour, respectively:

(2) $$\begin{array}{c} X_{B}^{j}=MultiHead\left(B^{j*},B^{j*},B^{j*}\right),j\in \left\{I,II,II\right\}\text{.} \end{array}$$

Finally, these three output matrices are stitched together into a three-dimensional array *X*_*B*_ ∈ *ℝ*^3×*n*×*d*_*v*_^, which represents the constraint in the same behaviours.

(3) $$\begin{array}{c} X_{B}=Concat\left(X_{B}^{I},X_{B}^{II}{,X}_{B}^{III}\right)\text{.} \end{array}$$

**Figure 5:**
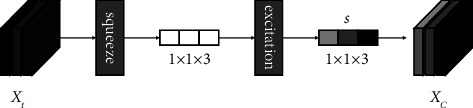
Modelling collaboration in different behaviours.

*(2) Modelling Collaboration in Different Behaviours*. It is also important to note that there is a mutual collaboration between multiple types of learning behaviours, i.e., the interaction of multiple types of learning behaviours in a learning sequence. Because the channel attention mechanism is able to capture the global information of multiple types of learning behaviours and translate it into the relative weights of each learning behaviour, the collaboration in different behaviours is modelled using the channel attention mechanism, as shown in Figure 5.

Using the array *X*_*t*_ as the input of the channel attention mechanism, the attention weights are obtained by collecting the global information of three types of learning behaviours to model collaboration in different behaviours, and the magnitude of the attention weights indicates the degree of collaboration of learning behaviours. The squeeze operation collects the global information of learning behaviours, and the excitation operation translates the above global information into attention weights *s* among different learning behaviours through a fully connected layer:

(4) $$\begin{array}{c} s=Sigmoid\left(W∙RC\left(Cov\left(X_{t}\right)\right)\right)\text{,} \end{array}$$

where Sigmoid=1∕(1+*e*^−*x*_*i*_^), the weight matrix of the fully connected layer is *W*, RC denotes the rowwise convolution, and Cov(∙) denotes the calculation of the covariance matrix, which is used to characterize the degree of correlation between the three types of learning behaviours.

The output attention weight *s* represents the collaboration in different behaviours, which is multiplied with the array *X*_*t*_ by the channel, changing the expression of the array *X*_*t*_ eigenvalues to obtain the array *X*_*C*_, representing the collaboration in different behaviours assigned to the array *X*_*t*_:

(5) $$\begin{array}{c} X_{C}=s∙X_{t}\text{.} \end{array}$$

The array *X*′ ∈ *ℝ*^6×*n*×*d*_*v*_^ is obtained by summing the array *X*_*B*_, which represents the constraint in the same behaviours, and the array *X*_*C*_, which represents the collaboration in different behaviours. By using the convolution kernel of 6 × 1 × *d*_*v*_, the dimension of array *X*′ is reduced by row-wise convolution to obtain the output matrix *X*_*E*_ ∈ *ℝ*^n×*d*_*v*_^:

(6) $$\begin{array}{c} X_{E}=RC\left(\left[X_{B}{,X}_{C}\right]\right)\text{.} \end{array}$$

The temporal convolutional networks (TCNs) model uses a 1D Fully Convolutional Networks (1D FCNs) structure to ensure that the input sequence and output of each hidden layer have the same length so that no matter which layer of the network, the input at each time has a corresponding output. In addition, TCN uses causal convolution to satisfy the feature that sequence data does not use future information, that is, when the model outputs the results at time *t*, it can only input data before time *t*.

Furthermore, the TCN model uses the derived calcium transformations to obtain longer historical information, avoiding the construction of deeper neural networks. For one-dimensional input sequence *X*={*x*_1_, *x*_2_,*x*_3_,…, *x*_*t*_}, convolution kernel *f*={0,1,2,…, *k* − 1}, the expansion convolution operation can be expressed as follows:

(7) $$\begin{array}{c} TCN\left(x_{t}\right)=\overset{k-1}{\underset{i=0}{\sum }}f\left(i\right)x_{t-ic}\text{,} \end{array}$$

where *c* is the expansion coefficient; *k* is the size of convolution kernel; *x*_*t*−*ic*_ represents the past data.

#### 3.3.3. Decoder

**Figure 6:**
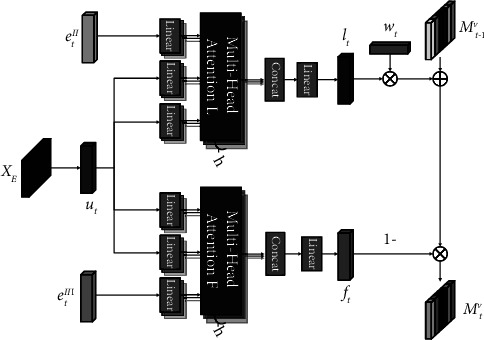
Decoder.

The decoder consists of two multi-headed attention mechanisms, which generate the learning vector and forgetting vector, respectively, through matrix *X*_*E*_. The structure is shown in Figure 6. Firstly, the *t* th learning end data *e*_*t*_^*II*^ is used as query input to represent the learning vector *l*_*t*_ in different dimensions; secondly, the *t* th learning interval data *e*_*t*_^*III*^ is used as query input to represent the forgetting vector *f*_*t*_ in different dimensions; finally, the state matrix *M*^*v*^ is updated according to the vectors *l*_*t*_ and *f*_*t*_.

The decoding vector *u*_*t*_ ∈ *ℝ*^1×*d*_*v*_^ is obtained by feeding the matrix *X*_*E*_ into the TCN to obtain. *u*_*t*_ represents the integration of constraints in the same behaviour and collaboration in different behaviours:

(8) $$\begin{array}{c} u_{t}=TCN\left(X_{E}\right)\text{.} \end{array}$$

The decoding vector *u*_*t*_ contains the constraint in the same behaviours and collaboration in different behaviours, which is used as the input to the key and value in the multi-headed attention mechanism *L* and *F* in Figure 6.

In the multiheaded attention mechanism *L*, the learning vector *l*_*t*_ is obtained using the vector *e*_*t*_^*II*^ as the query input:

(9) $$\begin{array}{c} l_{t}=\text{Tanh}\left(W_{l}^{T}Softmax\left(e_{t}^{II}u_{t}^{T}\right)u_{t}+b_{l}\right)\text{,} \end{array}$$

where Softmax(*x*_*i*_)=*x*_*i*_/∑_*n*=1_^*N*^(*ⅇ*^*x*_*n*_^), vector *e*_*t*_^*II*^ is the transformed learning end data, the vector describes the information of students' answer situation, and using it as the query input of decoding process can get the change of students' knowledge state due to the *t* th learning.

In the multiheaded attention mechanism *F*, the forgetting vector *f*_*t*_ is obtained with the vector *e*_*t*_^*III*^ as the query input:

(10) $$\begin{array}{c} f_{t}=Sigmoid\left(W_{f}^{T}Softmax\left(e_{t}^{III}u_{t}^{T}\right)u_{t}+b_{f}\right)\text{,} \end{array}$$

where vector *e*_*t*_^*III*^ is the processed learning interval data, which describes the learning behaviour such as the time interval between two adjacent learning sessions and the number of times a student learns a concept and using it as the query input of the decoding process can obtain the changes of the students' concept mastery status due to forgetting.

The learning vector *l*_*t*_ and the forgetting vector *f*_*t*_ and the associated weights *w*_*t*_ are used to update the concept state matrix at the current moment, the association weights *w*_*t*_ will be described in the prediction module:

(11) $$\begin{array}{c} M_{t}^{v}\left(i\right)=M_{t-1}^{v}\left(i\right)\left(1-f_{t}\right)+l_{t}w_{t}\left(i\right)\text{.} \end{array}$$

#### 3.3.4. Conceptual Attention Module

The conceptual attention module uses the self-attention mechanism to strengthen the relationship between concepts according to the similarity between concept vector representations. The more similar the concept vector representations are, the stronger the relationship is.

First, the exercise *q*_*t*_ is converted into one-hot encoding and multiplied with the embedding matrix *A* ∈ *ℝ*^*d*_*v*_×N^ to obtain the exercise embedding vector *k*_*t*_ with dimension *d*_*k*_, which describes the information related to the exercise *q*_*t*_.

Second, the self-attention mechanism is used to strengthen the connection between concepts with high similarity, and the output matrix *C* is obtained:

(12) $$\begin{array}{c} C_{t-1}=Attention\left({M_{t-1}^{k}}^{T}\times M_{t-1}^{k}\right)M_{t-1}^{k}\text{.} \end{array}$$

Finally, vector *k*_*t*_ is multiplied with the concept matrix *C* ∈ *ℝ*^*d*_*v*_×N^ that stores the concepts and transformed into the associated weights *w*_*t*_ by the Softmax function, which is used to describe the concepts contained in the exercise *q*_*t*_:

(13) $$\begin{array}{c} w_{t}=Softmax\left(k_{t}\times C_{t-1}\right)\text{.} \end{array}$$

#### 3.3.5. Prediction Module

The prediction module is used to predict students' future answers. First, the association weights *w*_*t*_ are multiplied with the state matrix *M*_*t*−1_^*v*^ to obtain the vector *n*_*t*_, which represents the student's mastery status of the concepts contained in exercise *q*_*t*_:

(14) $$\begin{array}{c} n_{t}=w_{t}M_{t-1}^{v}\text{.} \end{array}$$

Second, considering that there are certain differences between the exercises, such as different difficulty coefficients, the vector *n*_*t*_ is spliced with the vector *k*_*t*_ and fed into the fully connected layer with Tanh activation function to obtain the vector *i*_*t*_. The vector *i*_*t*_ contains both the student's mastery state of the concept and the information of the exercise:

(15) $$\begin{array}{c} i_{t}=\text{Tanh}\left(W_{2}^{T}\left[n_{t},k_{t}\right]+b_{2}\right)\text{.} \end{array}$$

Finally, an output layer with a Sigmoid activation function, using vector *i*_*t*_ as input, is used to predict student performance on the exercise *q*_*t*_:

(16) $$\begin{array}{c} P_{t}=Sigmoid\left(W_{3}^{T}i_{t}+b_{3}\right)\text{.} \end{array}$$

### 3.4. Loss Function

In this paper, the cross-entropy loss function is chosen to minimize the variability between the predicted value *P*_*t*_ and the true value *r*_*t*_:

(17) $$\begin{array}{c} Loss=-\underset{t}{\sum }\left(r_{t}\log P_{t}+\left(1-r_{t}\right)\log\;\left(1-P_{t}\right)\right)\text{.} \end{array}$$

## 4. Experiments and Analysis

### 4.1. Data Set and Experimental Environment

The experiments related to this paper are conducted on three real datasets: ASSISTments2012 (Assist12), ASSISTments2017 (Assist17), and Junyi Academy (Junyi). In each dataset, 70% of the data were used as a training set and 30% of the data were used as a test set. The basic information of the above datasets is shown in Table 1, including the number of students, the number of learning records, and the number of concepts.

The experiments in this paper are implemented under the Windows system with GeForce graphics acceleration units, based on python and PyTorch platforms, with the hardware and software configurations shown in Table 2.

### 4.2. Implementation Details

In each dataset, 80% of the data is divided into a training set and 20% of the data was divided into a test set. Twenty percent of the data in the training set was divided into the validation set, which was used to select the hyperparameters of the best model. Considering that the data sets differ in the number of learners, the number of exercise interactions, and the number of concepts, the learning rate was initialized to 0.001 and reduced by 10 every 10 epochs. Adam was chosen as the optimizer with the batch-size set to 32. The initialization of the parameters was chosen to be randomly initialized with a Gaussian distribution with zero mean and standard deviation.

The AED-KT model also focuses on the input set size *n*, the dimension *d*_*v*_ of the state matrix, the dimension *d*_*k*_ of the state matrix, and the expansion coefficient of TCN *c*. To facilitate the calculation, set *d*=*d*_*v*_=*d*_*k*_. In the dataset ASSISTments2012, the input set size *n* was set to 32, the dimension *d* was set to 64, and the expansion coefficient *c*={1,2,4,8,16}. In the dataset ASSISTments2017, the input set size *n* was set to 32, the dimension *d* was set to 64, and the expansion coefficient *c*={1,2,4,8,16}. In the dataset Junyi, the input set size *n* was set to 32, the dimension *d* was set to 64, and the expansion coefficient *c*={1,2,4,8,16}.

### 4.3. Evaluating Indicator and Baseline

#### 4.3.1. Evaluating Indicator

The performance of the AED-KT model proposed in this paper is analysed and evaluated using the metric Area Under Curve (AUC), which is the area of the graph enclosed by the ROC curve and the horizontal axis, and the value of this area is between 0.5 and 1. If the value of AUC is 0.5, it means that the model is a stochastic prediction model; the larger the value of AUC, the better the prediction performance of the model.

#### 4.3.2. Baseline

The core of the AED-KT model is to use three types of learning behaviours as input and model the constraint in the same behaviours and collaboration in different behaviours using the multihead and channel attention mechanisms, respectively. Based on the above-mentioned theory, we mainly consider the following three conditions when selecting the comparison model: first, the comparison model belongs to the widely accepted model with the best-in-class performance, second, the type of input learning behaviours data of the comparison model, and third, the comparison methods model constraint in same behaviours and collaboration in different behaviours. Based on the above-mentioned three conditions, we choose the following model as the comparison model:

  DKT [3]: DKT is the first time to use the deep learning method in the field of knowledge tracing, using high-dimensional vectors in RNN or LSTM to represent the students' knowledge state. But there are problems of long sequence dependency and gradient explosion, and students' specific mastery of each concept cannot be obtained according to the vector.
  DKVMN [4]: DKVMN uses a static matrix to store concepts and a dynamic matrix to store knowledge states. Thanks to this design, DKVMN is able to know the student's knowledge state for each concept, solving the problem that DKT uses a hidden state to represent the student's overall knowledge state. But without the long-term dependence of modelling sequence data.
  SAKT [24]: SAKT is based on the Transformer model to accomplish the knowledge tracing task. Thanks to the Transformer architecture, the SAKT model can be trained in parallel, solving the problem that recurrent neural networks cannot be trained in parallel.
  DKT-F [6]: DKT-F models forgetting behaviour based on the DKT model by introducing learning interval data as input, but the forgetting mechanism is not interpretable.
  DKT-DT [5]: DKT-DT introduces learning process data as input on the basis of DKT, while using the decision tree method to analyse learning process data and feature selection on learning process data, the model can analyse richer feature energy and judge students' knowledge status more effectively.
  TCN-KT [31]: TCN-KT used LSTM to model the students' prior basis, combined with temporal convolution neural network to complete the knowledge tracing task, and solved the problem that DKT could not obtain long-term dependence.

The main reason is that all of these models take as input some or all of the learning behaviours data and model constraint in the same behaviours or collaboration in different behaviours. Specifically, DKT, DKVMN, and TCN-KT use learning end data as input and model constraint in the same behaviours using a sequence model; SAKT also uses learning end data as input and also models constraint in the same behaviours using a multiheaded attention mechanism; DKT-F and DKT-DT add learning interval and learning process data, respectively, as input, in addition to using learning end data. The constraint in the same behaviours is modelled using the sequence model, and collaboration in different behaviours is not modelled.

### 4.4. Model Performance Comparison

The results of the performance comparison experiments are shown in Table 3.

The above-mentioned models can be divided into two categories: single learning behaviour models, which refer to models that use only learning end data as input and multiple learning behaviours models, which refer to models that use learning end data as input and introduce other learning behaviours data.

The single learning behaviour models are mainly DKT, DKVMN, and SAKT. Among them, the AUC values of SAKT reached 0.734 and 0.853 on the Assist17 and Junyi datasets, which are the highest of their kind and have good overall performance. Although all three models use learning end data as input, the differences in modelling constraint in the same behaviours lead to differences in model performance.

The main multilearning behaviours models are DKT-F, DKT-DT, and AED-KT. The first two models introduce other learning behaviours data as inputs, which do not improve the method of modelling collaboration in different behaviours but improve the inputs of the models, and all perform better compared with single learning behaviour models. The AUC values of AED-KT outperform the other models on all three datasets. This illustrates the effectiveness of modelling collaboration in different behaviours based on modelling constraint in the same behaviours.

### 4.5. Training Process Comparison

We use the early stop strategy to compare the number of training rounds required for the model to reach the same performance. Using the early stop strategy can avoid overfitting the model during training. To verify the impact of using the early stopping strategy, we trained 200 epochs for AED-KT. The experimental results are shown in Table 4.

Table 4 shows that with the early stop strategy, the deviation between the AUC of the Asisstment2012 training set and the ASSISTments2012 test set is not large, and there is no fitting phenomenon. However, after 200 epochs of training the model directly, there is a large deviation between the AUC of the Asisstment2012 training set and the Asisstment2012 test set, and there is a problem of overfitting. In addition, the AUC of the training model with an early stop strategy is 0.768 on the Asisstment2012 training set, and the AUC of the model with 200 training cycles is 0.757. The difference is in a reasonable range, and there is no obvious underfitting phenomenon.

**Figure 7:**
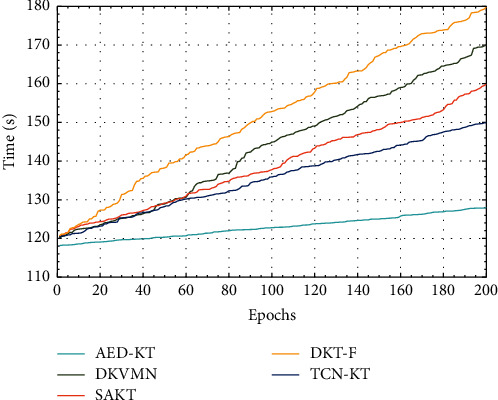
Time cost.

Furthermore, we explored the time cost of AED-KT and the comparison models when training the same epochs, and the experimental results are shown in Figure 7.

As can be seen from Figure 7, the time cost of AED-KT and TCN-KT training is lower than that of DKVMN, DKT-F, and SAKT. This is because AED-KT and TCN-KT use TCN to build models. TCN does not need to process data in time sequence to the recurrent neural network, which reduces the time cost. Compared with TCN-KT, AED-KT costs less time. This is because TCN-KT first uses LSTM to model students' prior basis, and then models their knowledge level.

### 4.6. Comparison of Learning Behaviours

The model performance comparison results show that the introduction of other learning behaviours as inputs can lead to improved model performance. To further compare and analyse the importance of the three types of learning behaviours in the model, we adjust the inputs of the default AED-KT model: AED-e indicates that the model uses only learning end data *b*^*II*^ as input; AED-pe indicates that the model uses learning process data *b*^*I*^ and learning end data *b*^*II*^ as input; AED-ei indicates that the model uses learning end data *b*^*II*^ and learning interval data *b*^*III*^ as input. The above-given models and their AUC values on the three datasets are given in Table 5.

The experimental results show that the AUC values of the AED-e model are lower than those of the other models on the three real data sets, indicating that analysing only the constraint in learning end behaviour can basically determine the students' knowledge status, but since *b*^*II*^ only contains learning end data of students' correct or incorrect answers, it contains limited information and cannot model the constraint in the same behaviours more accurately. The AED-pe and AED-ei models have higher AUC values than AED-e, indicating that introducing other learning behaviours data as input and modelling both constraint in the same behaviours and collaboration in different behaviours can improve the performance of the model on the basis of using learning end data as input; however, the AUC values of these two models are lower than AED-KT, indicating that on the basis of modelling both constraint in same behaviours and collaboration in different behaviours. However, the AUC values of these two models are lower than those of AED-KT, indicating that the more comprehensive the learning behaviours analysed by the model, the higher the performance of the model.

### 4.7. Encoder Ablation Experiment

To analyse modelling constraint in the same behaviours and collaboration in different behaviours impact on model performance, we ablation the attention mechanisms in the encoders that model the constraint in the same behaviours and collaboration in different behaviours: the model in the AED-B representation models only the constraint in same behaviours, and the model in the AED-C representation models only collaboration in different behaviours. The above-given models and their AUC values on the three datasets are given in Table 6.

The experimental results show that the AUC values of the AED-KT model are better than the other two models on all three data sets, indicating that it is effective to analyse the constraint in the same behaviours and collaboration in different behaviours when considering the three types of learning behaviours together. The AUC values of the AED-C model are lower than those of the AED-B model on the three datasets, indicating that modelling only the collaboration in different behaviours while ignoring the similarity constraint will result in the model losing the constraint relationship of learning behaviours on the time series and will not improve the performance of the model. The AUC values of the AED-B model on the three real data sets is lower than those of the AED-KT model, indicating that modelling only the constraint in the same behaviours and ignoring the collaboration in different behaviours leads to the loss of the interactions of multiple types of learning behaviours, which also fails to improve the performance of the model.

### 4.8. Comparison of Model Representation Quality

The representation quality of a model refers to the overall difference between the predicted and actual results of the model in a real application. For example, the knowledge tracing model KT is trained from a real data set and has good predictive performance. When the model KT is applied to a real teaching environment if the model predicts that 40% of the students will answer the questions about concept C incorrectly, but the actual results show that only 10% of the students answer the questions about concept C incorrectly, this indicates that the overall difference between the prediction results and the actual results of the model KT in practice is large and the representation quality of the model needs to be improved.

The model KT in the above example has good predictive performance but performs poorly in practice and cannot be applied in a real teaching environment, indicating that both high predictive performance and the quality of the representation of the model are critical. The consistency between predicted and observed probabilities is generally measured using calibration curves [35], and the representation quality of each model is measured using the baseline alignment line *x*=*y*. A calibration curve that is closer to the baseline alignment line indicates that the model prediction probability is closer to the observed probability, i.e., the model has better representation quality.  Table 7 shows the position of the calibration curve of each model in relation to the baseline (Assist12 is used as an example).

According to Table 7, first, the AED-KT model calibration curve value to the baseline at both lower and higher Prediction probability, indicating that the AED-KT model has better representation quality in comparison with the comparison model. Second, although the calibration curve value of the SAKT model is also close to the baseline, the calibration curve value of this model shows more dramatic fluctuations than that of the AED-KT model, i.e., the representation quality of the SAKT model is unstable on the whole. The results in Table 7 show that AED-KT is effective in considering multiple learning behaviours and modelling the constraint in the same behaviours and collaboration in different behaviours, which enables the model to not show severe bias and to obtain better representation.

## 5. Conclusions

In this paper, we propose AED-KT, a knowledge tracing model with multiple learning behaviours, to solve the problem that the existing knowledge tracing models cannot accurately describe the boundedness of a single type of learning behaviour in time series; or cannot accurately describe the interaction of multiple types of learning behaviours. AED-KT model uses a multiheaded attention mechanism to represent the constraint in the same behaviours and uses a channel attention mechanism to represent the collaboration in different behaviours. Fusing the constraint in the same behaviours and the collaboration in different behaviours to complete the synergistic representation of different types of learning behaviours. The experimental results of the proposed knowledge tracing model and five comparison models on three real datasets show that the proposed AED-KT model performs better and validates the effectiveness of the constraint in the same behaviours and collaboration in different behaviours. In the future, we will continue to investigate the impact of the constraint in the same behaviours and collaboration in different behaviours on the knowledge tracing model in depth.

## Figures and Tables

**Table 1:** Datasets information.

Data set	Learners	Records	Concepts
Assist12	46674	5818868	266
Assist17	1709	942816	102
Junyi	238120	26666117	684

**Table 2:** Experiment environment.

Experimental configuration	Parameter value
Operating system	Windows 11
CPU	Inter(R) core(TM) i9-9900K CPU@3.60 GHz
GPU	NVIDIA GeForce RTX 3080 Ti
Python	3.10
PyTorch	1.10.2
RAM	64 GB

**Table 3:** Model performance comparison.

Model	Date set		
	Assist12	Assist17	Junyi
DKT	0.717 [32]	0.726 [33]	0.814 [34]
DKVMN	0.732 [34]	0.707 [33]	0.822 [34]
SAKT	0.691	0.734 [24]	0.853
DKT-F	0.722 [34]	0.729	0.840 [34]
DKT-DT	0.749	0.721	0.741 [5]
TCN-KT	0.743	0.732	0.758
AED-KT	0.768 ± 0.003	0.815 ± 0.005	0.864 ± 0.004

**Table 4:** Experimental results of different training strategies.

Method	Assist12 training set	Assist12 testing set
Early stop	0.768	0.757
200 epochs	0.757	0.729

**Table 5:** The impact of different data on model performance.

Model	Data set		
	Assist12	Assist17	Junyi
AED-e	0.724 ± 0.004	0.778 ± 0.007	0.829 ± 0.005
AED-pe	0.763 ± 0.005	0.805 ± 0.006	0.856 ± 0.004
AED-ei	0.761 ± 0.004	0.799 ± 0.006	0.844 ± 0.006
AED-KT	0.768 ± 0.003	0.815 ± 0.005	0.864 ± 0.004

**Table 6:** Encoder ablation experiment.

Model	Data set		
	Assist12	Assist17	Junyi
AED-B	0.760 ± 0.007	0.806 ± 0.008	0.859 ± 0.007
AED-C	0.757 ± 0.005	0.788 ± 0.009	0.843 ± 0.006
AED-KT	0.768 ± 0.003	0.815 ± 0.005	0.864 ± 0.004

**Table 7:** Calibration curve value.

Model	Prediction probability								
Baseline	0.15	0.18	0.23	0.39	0.45	0.58	0.65	0.75	0.85
AED-KT	0.154	0.185	0.233	0.396	0.455	0.584	0.652	0.754	0.855
DKT	0.113	0.152	0.201	0.353	0.425	0.554	0.622	0.728	0.829
DKVMN	0.123	0.164	0.215	0.361	0.438	0.567	0.634	0.739	0.837
SAKT	0.130	0.165	0.222	0.373	0.436	0.573	0.642	0.741	0.844
DKT-F	0.161	0.194	0.242	0.400	0.459	0.594	0.664	0.764	0.863
DKT-DT	0.173	0.198	0.255	0.405	0.467	0.600	0.671	0.771	0.871
TCN-KT	0.165	0.181	0.251	0.412	0.468	0.597	0.673	0.780	0.864

## Data Availability

The data used to support the findings of this study can be obtained from the corresponding author upon request.
